# Circ_0043947 contributes to interleukin 1β-induced injury in chondrocytes by sponging miR-671-5p to up-regulate RTN3 expression in osteoarthritis pathology

**DOI:** 10.1186/s13018-022-02970-4

**Published:** 2022-03-24

**Authors:** Min He, Zhihe Jia, Yiying Wen, Xiaolin Chen

**Affiliations:** 1 Department of Joint Surgery, Pingxiang People’s Hospital, Pingxiang City, 337055 Jiangxi China; 2 Department of Laboratory, Pingxiang People’s Hospital, No. 8 Wugong Shanzhong Avenue, Development Zone, Pingxiang City, Jiangxi China

**Keywords:** Osteoarthritis, IL-1β, circ_0043947, miR-671-5p, RTN3

## Abstract

**Objective:**

Osteoarthritis (OA) is a chronic joint disease featured by articular cartilage degeneration and damage. Accumulating evidence have demonstrated the pivotal regulatory roles of circular RNAs in OA pathology. However, the role of circ_0043947 in OA progression and its associated mechanism remain largely unknown.

**Methods:**

The expression of RNA and protein was determined by reverse transcription-quantitative polymerase chain reaction and Western blot assay. Cell viability was assessed by 3-(4,5-Dimethylthiazol-2-yl)-2,5-diphenyltetrazolium bromide (MTT) assay. Cell proliferation was analyzed by 5-Ethynyl-2′-deoxyuridine (EdU) assay and flow cytometry. Cell apoptosis was assessed by flow cytometry. Enzyme linked immunosorbent assay was conducted to analyze the release of pro-inflammatory cytokines. Dual-luciferase reporter assay and RNA immunoprecipitation assay were performed to confirm the target interaction between microRNA-671-5p (miR-671-5p) and circ_0043947 or reticulon 3 (RTN3).

**Results:**

Interleukin 1β (IL-1β) stimulation up-regulated the expression of circ_0043947 in chondrocytes. IL-1β treatment restrained the viability and proliferation and induced the apoptosis, extracellular matrix degradation and inflammatory response of chondrocytes partly by up-regulating circ_0043947. Circ_0043947 interacted with miR-671-5p, and miR-671-5p silencing largely reversed circ_0043947 knockdown-mediated protective effects in IL-1β-induced chondrocytes. miR-671-5p interacted with the 3′ untranslated region (3′UTR) of RTN3. miR-671-5p overexpression attenuated IL-1β-induced injury in chondrocytes, and these protective effects were largely overturned by the overexpression of RTN3. Circ_0043947 acted as a molecular sponge for miR-671-5p to up-regulate RTN3 level in chondrocytes.

**Conclusion:**

Circ_0043947 silencing alleviated IL-1β-induced injury in chondrocytes by targeting miR-671-5p/RTN3 axis.

## Introduction

Osteoarthritis (OA) is a common degenerative joint disease involving the disease processes of all joint tissues, including cartilage and meniscal degeneration, subchondral bone remodeling, synovial inflammation and inflammation and fibrosis of the infrapatellar fat pad [1–4]. Several factors, including genetics, ageing, repetitive joint injury, obesity, metabolic disorders and anatomical factors related to joint shape and alignment, may initiate OA pathology [5, 6]. In recent years, the central role of inflammation in OA has been gradually discovered [7]. Interleukin-1β (IL-1β) is a vital pro-inflammatory factor, which promotes the release of matrix metalloproteinases (MMPs) into the articular cavity and induces cartilage degradation [8]. Therefore, inhibition of these inflammatory mediators may be a potential therapeutic method for OA patients.

Circular RNAs (circRNAs) are characterized by stable circular structure that formed by back-splicing at the 5′ and 3′ terminals [9]. Recently, accumulating articles have pointed out the vital functions of circRNAs in the pathology of human diseases due to their regulatory roles in gene expression [10, 11]. Previous study has shown that multiple circRNAs are dysregulated in OA tissues compared with normal cartilage [12]. Wu et al. demonstrated that circ_0005105 contributes to extracellular matrix (ECM) degradation through mediating microRNA-26a (miR-26a)/NAMPT axis in OA [13]. In this study, we evaluated the role and working mechanism of a novel circRNA, circ_0043947, in OA pathology using IL-1β-induced chondrocytes.

It is generally accepted that circRNAs can serve as competing endogenous RNAs (ceRNAs) to competitively bind to miRNAs, thereby leading to the up-regulation of the downstream genes [14]. Increasing studies have associated dysregulated miRNAs with multiple pathological changes in OA, including articular cartilage destruction and chondrocyte apoptosis [15, 16]. starbase and circinteractome bioinformatics databases predicted the potential interaction between circ_0043947 and miR-671-5p. Previous studies reported that miR-671 suppresses OA development and attenuates IL-1β-induced damage in chondrocytes [17, 18]. In this study, we tested the target relationship between circ_0043947 and miR-671-5p and further explored their functional relevance in OA progression.

miRNAs can induce the degradation or translational repression of mRNAs by interacting with their 3′ untranslated region (3′UTR) [19]. Through starbase bioinformatics database, we found that the 3′UTR of reticulon 3 (RTN3) was complementary with miR-671-5p. Fu et al. demonstrated that LINC02288 promotes the apoptosis and inflammatory response of chondrocytes partly by up-regulating RTN3 via absorbing miR-374a-3p [20]. In this study, we analyzed the target relationship between RTN3 and miR-671-5p and further explored their functional association in OA pathology.

In this study, we explored the important molecules and signal pathways involved in OA pathology using IL-1β-induced chondrocytes as cell model. The role of circ_0043947 in regulating the phenotypes of IL-1β-induced chondrocytes was initially analyzed. Then, the downstream mechanism of circ_0043947 was explored by bioinformatics analysis and functional experiments.

## Materials and methods

### Cell culture

Human primary chondrocytes (isolated from healthy subjects) were purchased from Procell (Wuhan, China). Chondrocytes were cultured in chondrocyte complete medium (Procell) added with 1% penicillin/streptomycin mixture (Sangon Biotech, Shanghai, China) at 37 °C with 5% CO_2_. Human embryonic kidney HEK293T cells were purchased from Shanghai Academy of Sciences (Shanghai, China) and were cultured in Dulbecco’s modified Eagle’s medium (DMEM) (Hyclone, Logan, UT, USA) supplemented with 10% fetal bovine serum (FBS; Hyclone) and 1% penicillin/streptomycin mixture (Sangon Biotech) at 37 °C with 5% CO_2_.

### RNase R and Actinomycin D (Act D) treatment

The circular structure and stability of circ_0043947 were tested using exonuclease RNase R and transcriptional inhibitor Act D, respectively.

RNA samples were treated with RNase R (100 μg/mL; Applied Biological Materials, Vancouver, Canada) for 20 min. Then, RT-qPCR analysis was performed to determine RNA levels. Chondrocytes were incubated with Act D (2 mg/mL; Sigma, St. Louis, MO, USA) for 4 h, 8 h or 12 h, and RT-qPCR was conducted to analyze RNA levels.

### IL-1β stimulation

Chondrocytes were seeded onto 6-well plates at the density of 2.0 × 10^5^ cells/well. Chondrocytes were stimulated with 10 ng/mL IL-1β (PeproTech, Suzhou, China) for 24 h. The treated chondrocytes were utilized for further analysis.

### Reverse transcription-quantitative polymerase chain reaction (RT-qPCR)

Chondrocytes were seeded onto 6-well plates at the density of 2.0 × 10^5^ cells/well. After the indicated treatment, RNA samples were isolated with Trizol reagent (Thermo Fisher Scientific, Foster City, CA, USA). The reverse transcription was conducted using a commercial RevertAid First-Strand complementary DNA (cDNA) Synthesis kit (Thermo Fisher Scientific) and stem-loop reverse-transcription universal primer (Ribobio, Guangzhou, China). cDNA was amplified using RT-qPCR mix (Thermo Fisher Scientific) and specific primers (Table 1). The fold changes were assessed by the 2 (-Delta Delta C(T)) method [21] with the references of glyceraldehyde-phosphate dehydrogenase (GAPDH; for circRNAs and mRNAs) and U6 (for miRNAs).

**Table 1:** Primer sequences in RT-qPCR

Gene	Forward primer (5′–3′)	Reverse primer (5′–3′)
circ_0043947	ATCATTCACCCTTGGCACAG	CATTTTCCTCCCGCAATTC
BRCA1	TTCACCCTCTGCTCTGGGTA	TGGTCACACTTTGTGGAGACA
miR-671-5p	GCCGAGAGGAAGCCCTGGAGGGG	CTCAACTGGTGTCGTGGA
RTN3	CATCTCCTCGTCGTCCTTCG	AATCAGATCGTGCACCGCA
U6	CTCGCTTCGGCAGCACA	AACGCTTCACGAATTTGCGT
GAPDH	GAAAGCCTGCCGGTGACTAA	TTCCCGTTCTCAGCCTTGAC

### Cell transfection

Small interfering (si)RNAs against circ_0043947 (circ_0043947-si#1 and circ_0043947-si#2), the matched negative control (si-NC), mimics of miR-671-5p (miR-671-5p), NC of miRNA (miR-NC), inhibitor of miR-671-5p (anti-miR-671-5p), anti-miR-NC, RTN3 overexpression plasmid (RTN3) and pcDNA control vector (pcDNA) were purchased from GenePharma (Shanghai, China). Transient transfection was performed with Lipofectamine 3000 reagent (Invitrogen, Carlsbad, CA, USA).

### 3-(4,5-Dimethylthiazol-2-yl)-2,5-diphenyltetrazolium bromide (MTT) assay

Chondrocytes were seeded onto 96-well plates at the density of 3 × 10^3^ cells/well. After transfecting with RNAs or plasmids and exposing to IL-1β, chondrocytes were incubated with MTT reagent (Sangon Biotech) for 4 h. Afterward, dimethyl sulfoxide (DMSO; Solarbio, Beijing, China) was added to dissolve the formazan product, and the absorbance at 570 nm was examined using the microplate reader (Bio-Rad, Hercules, CA, USA).

### 5-Ethynyl-2′-deoxyuridine (EdU) assay

EdU assay was conducted to analyze cell proliferation ability using KeyFluor488 Edu Kit (keyGEN Biotech, Jiangsu, China). Chondrocytes were seeded onto 24-well plates at 5 × 10^4^ cells/well. Cell nucleus was stained with 4,6-diamino-2-phenyl indole (DAPI; Sigma). The fluorescence intensities were determined under the fluorescence microscope (Olympus, Tokyo, Japan). The representative images were captured at the magnification of 100×.

### Flow cytometry

Flow cytometry was conducted to analyze cell cycle progression and apoptosis of chondrocytes.

Chondrocytes were seeded onto 6-well plates at the density of 2.0 × 10^5^ cells/well. Chondrocytes were processed using Muse Cell Cycle Assay Kit (Millipore, Billerica, MA, USA), and the percentages of chondrocytes in G0/G1, S, and G2/M phases were assessed using flow cytometer (BD Biosciences, San Jose, CA, USA).

Chondrocytes were seeded onto 6-well plates at the density of 2.0 × 10^5^ cells/well. Chondrocytes were processed using commercial cell apoptosis kit (BD Biosciences). Chondrocytes were simultaneously stained with fluorescein isothiocyanate (FITC)-conjugated Annexin V and propidium iodide (PI) at room temperature for 15 min in the dark, and the percentage of chondrocytes with FITC^+^ and PI^±^ was analyzed using flow cytometer (BD Biosciences).

### Western blot assay

Chondrocytes were seeded onto 6-well plates at the density of 2.0 × 10^5^ cells/well. After the indicated treatment, cells were disrupted with radio-immunoprecipitation assay (RIPA) buffer (Beyotime, Jiangsu, China), and protein samples were quantified using the commercial bicinchoninic acid assay kit (Beyotime, Shanghai, China). Protein samples (25 μg/lane) were separated by sodium dodecyl sulfate–polyacrylamide gel electrophoresis (SDS-PAGE), and the protein bands were then blotted onto the polyvinylidene difluoride (PVDF) membrane (Bio-Rad). The membrane was incubated with 5% skimmed milk for 1 h at room temperature to block the non-specific sites. The membrane was incubated with diluted primary antibodies (Table 2) overnight at 4 °C. Subsequently, the membrane was incubated with horseradish peroxidase (HRP)-labeled secondary antibody (dilution: 1:5000; Abcam, Cambridge, MA, USA) for 2 h at room temperature. Protein signals were appeared using the electrochemiluminescence detection system (Thermo Fisher Scientific). The intensities of protein bands were quantified by the Image Lab analysis software (Bio-Rad).

**Table 2:** Primary antibodies in Western blot assay

Antibody name	Catalogue number	Dilution	Supplier
PCNA	ab29	1:8000	Abcam, Cambridge, MA, USA
Cyclin D1	ab40754	1:10,000	Abcam, Cambridge, MA, USA
c-caspase 3	ab32042	1:5000	Abcam, Cambridge, MA, USA
MMP3	ab52915	1:10,000	Abcam, Cambridge, MA, USA
MMP13	ab51072	1:3000	Abcam, Cambridge, MA, USA
Collagen II	ab188570	1:10,000	Abcam, Cambridge, MA, USA
RTN3	ab68328	1:3000	Abcam, Cambridge, MA, USA
GAPDH	ab8245	1:20,000	Abcam, Cambridge, MA, USA

PCNA, proliferating cell nuclear antigen; c-caspase 3, cleaved caspase 3; MMP3, matrix metallopeptidase 3; MMP13, matrix metallopeptidase 13

### Enzyme linked immunosorbent assay (ELISA)

The concentrations of interleukin 6 (IL-6), IL-8 and tumor necrosis factor α (TNF-α) in the culture supernatant were assessed using the ELISA kit (Elabscience Biotechnology, Wuhan, China).

### Establishment of circRNA/miRNA/mRNA axis

Bioinformatics databases, including starbase (http://starbase.sysu.edu.cn) and circinteractome (https://circinteractome.irp.nia.nih.gov), were utilized to establish circ_0043947-miRNAs interactions, whereas miR-671-5p-mRNAs interactions were established using starbase database.

### Dual-luciferase reporter assay

The linear sequence of circ_0043947 or the 3′ untranslated region (3′UTR) of RTN3, including the putative binding sites with miR-671-5p, was inserted into pGL3 plasmid (Promega, Fitchburg, WI, USA). The above sequences with mutant miR-671-5p binding sites were also cloned into pGL3 plasmid (Promega). The re-constructed plasmids were named as pGL3-circ_0043947 WT, pGL3-circ_0043947 MUT, pGL3-RTN3 3′UTR WT and pGL3-RTN3 3′UTR MUT. HEK293T cells were seeded onto 24-well plates at 5 × 10^4^ cells/well and were co-transfected with 100 ng luciferase reporter plasmids and 50 nM oligonucleotides. After transfection for 48 h, the changes in luciferase activities were determined using the Dual Glo Luciferase Assay System (Promega). The luciferase activity of Firefly was normalized to Renilla luciferase activity.

### RNA immunoprecipitation (RIP) assay

Chondrocytes were seeded onto 6-well plates at the density of 2.0 × 10^5^ cells/well and were transfected with miR-NC or miR-671-5p. At 48 h after transfection, RIP assay was conducted using Magna RIP RNA-Binding Protein Immunoprecipitation Kit (Millipore). Chondrocytes were disrupted with RIP lysis buffer for 5 min on the ice. RNA samples were then incubated with antibody-coated magnetic beads. The antibody against Argonaute2 (AGO2) or Immunoglobulin G (IgG) was purchased from Abcam. RT-qPCR was implemented to analyze RNA enrichment.

### Statistical analysis

The data were analyzed by GraphPad Prism 7.0 software (GraphPad, La Jolla, CA, USA) and were expressed as mean ± standard deviation (SD). The comparisons were analyzed using Student’s *t*-test (in two groups) or one-way analysis of variance (ANOVA) followed by Tukey’s test (in multiple groups). *P* < 0.05 was considered as statistically significant.

## Results

### IL-1β up-regulates circ_0043947 expression in chondrocytes

**Fig. 1:**
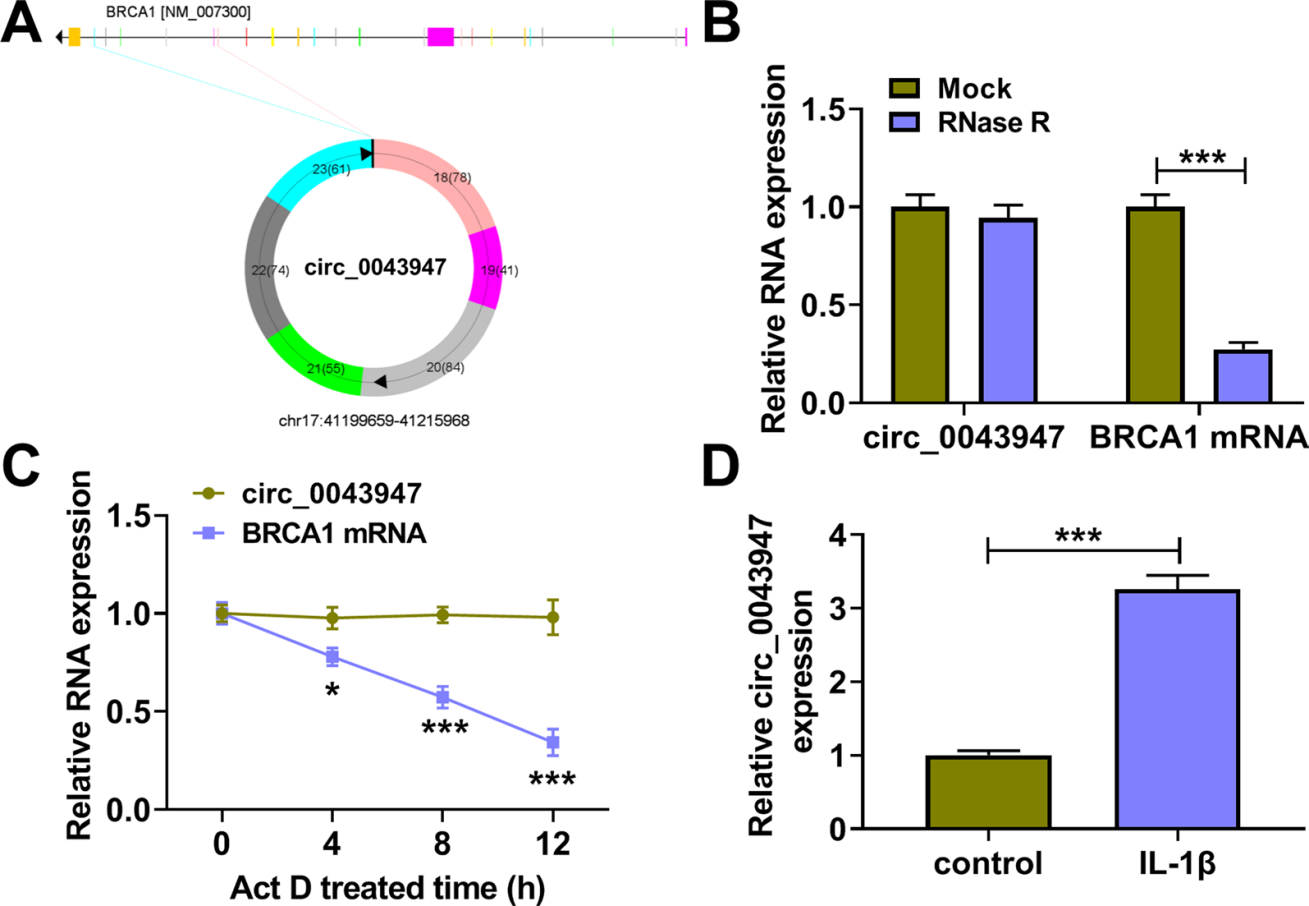
IL-1β up-regulates circ_0043947 expression in chondrocytes. **A** Circ_0043947 (393 nt) was derived from the back-splicing of exon 18–23 of BRCA1 gene. **B** The circular structure of circ_0043947 was tested by RNase R, and linear BRCA1 mRNA acted as the control. **C** The stability of circ_0043947 was examined by Act D, and linear BRCA1 mRNA served as the control. **D** The expression of circ_0043947 in IL-1β-induced chondrocytes (10 ng/mL, 24 h) was determined by RT-qPCR. *P < 0.05, ***P < 0.001

Circ_0043947 (393 nt) was generated from the back-splicing of exon 18–23 of its host gene breast cancer 1, early onset (BRCA1) (Fig. 1A). RNase R digestion significantly reduced the level of BRCA1 mRNA, while the level of circ_0043947 remained almost unchanged in RNase R group and Mock group (Fig. 1B), suggesting that circ_0043947 was a circular transcript without 5′ or 3′ end. Subsequently, transcriptional inhibitor Act D was used to analyze the stability of circ_0043947 and BRCA1 mRNA. We found that circ_0043947 was more stable than its linear form BRCA1 (Fig. 1C). To explore the role of circ_0043947 in OA progression in vitro, we established OA cell model by exposing chondrocytes to IL-1β (10 ng/mL; 24 h). We found that IL-1β treatment markedly up-regulated the expression of circ_0043947 in chondrocytes (Fig. 1D). Overall, circ_0043947 might be implicated in the regulation of OA progression.

### Circ_0043947 silencing protects chondrocytes against IL-1β-induced injury

**Fig. 2:**
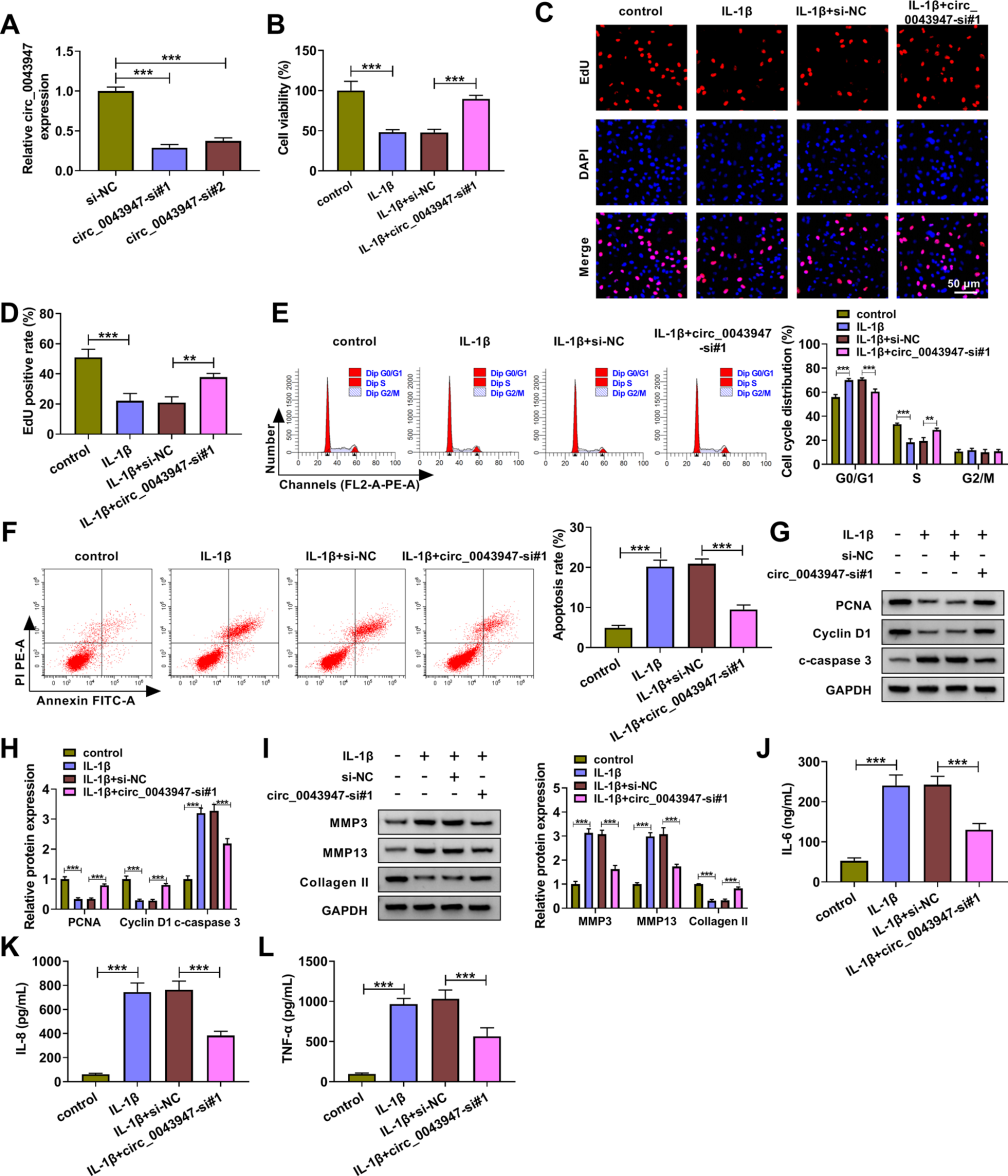
Circ_0043947 silencing protects chondrocytes against IL-1β-induced injury. **A** The transfection efficiencies of circ_0043947-si#1 and circ_0043947-si#2 in chondrocytes were determined by RT-qPCR. **B**-**L** Chondrocytes were transfected with si-NC or circ_0043947-si#1 followed by IL-1β (10 ng/mL) exposure for 24 h. **B** Cell viability was analyzed by MTT assay. **C** and **D** EdU assay was applied to analyze cell proliferation ability. **E** Cell cycle progression was assessed by flow cytometry. **F** Flow cytometry was performed to analyze cell apoptosis. **G** and **H** The expression of proliferation-associated proteins (PCNA and Cyclin D1) and apoptosis-associated protein (c-caspase 3) was determined by Western blot assay. **I** The levels of matrix-degrading enzymes (MMP3 and MMP13) and Collagen II were measured by Western blot assay. **J**-**L** The levels of inflammatory cytokines (IL-6, IL-8 and TNF-α) in culture supernatant were measured by ELISA. **P < 0.01, ***P < 0.001

Transfection with circ_0043947-si#1 or circ_0043947-si#2 markedly reduced circ_0043947 expression in chondrocytes, especially in circ_0043947-si#1 group (Fig. 2A), thus we selected circ_0043947-si#1 for the subsequent loss-of-function experiments. IL-1β stimulation decreased the viability of chondrocytes, and cell viability was largely recovered by the silence of circ_0043947 (Fig. 2B). EdU assay and flow cytometry were conducted to analyze the proliferation ability of chondrocytes. We found that IL-1β suppressed the proliferation of chondrocytes, and the silence of circ_0043947 largely rescued the proliferation ability of chondrocytes (Fig. 2C–E). IL-1β stimulation induced the apoptosis of chondrocytes, and cell apoptosis was notably suppressed by circ_0043947 interference (Fig. 2F). Western blot assay showed that circ_0043947 interference rescued the levels of proliferation-associated proteins (proliferating cell nuclear antigen (PCNA) and Cyclin D1) and reduced the level of pro-apoptotic cleaved caspase 3 (c-caspase 3) (Fig. 2G, H), which further demonstrated that circ_0043947 interference promoted the proliferation and suppressed the apoptosis of IL-1β-treated chondrocytes. The main component of ECM (Collagen II) and two matrix-degrading enzymes (MMP3 and MMP13) were determined by Western blot assay. The results revealed that circ_0043947 interference reduced the levels of MMP3 and MMP13 whereas increased Collagen II expression in IL-1β-treated chondrocytes (Fig. 2I), indicating that circ_0043947 interference protected chondrocytes from IL-1β-induced ECM degradation. Cell inflammatory response was also assessed. IL-1β treatment promoted the production of inflammatory cytokines (IL-6, IL-8 and TNF-α), and cell inflammation was restrained by circ_0043947 interference (Fig. 2J–L). These results demonstrated that IL-1β-induced injury in chondrocytes was largely based on the up-regulation of circ_0043947.

### Circ_0043947 acts as a molecular sponge for miR-671-5p

**Fig. 3:**
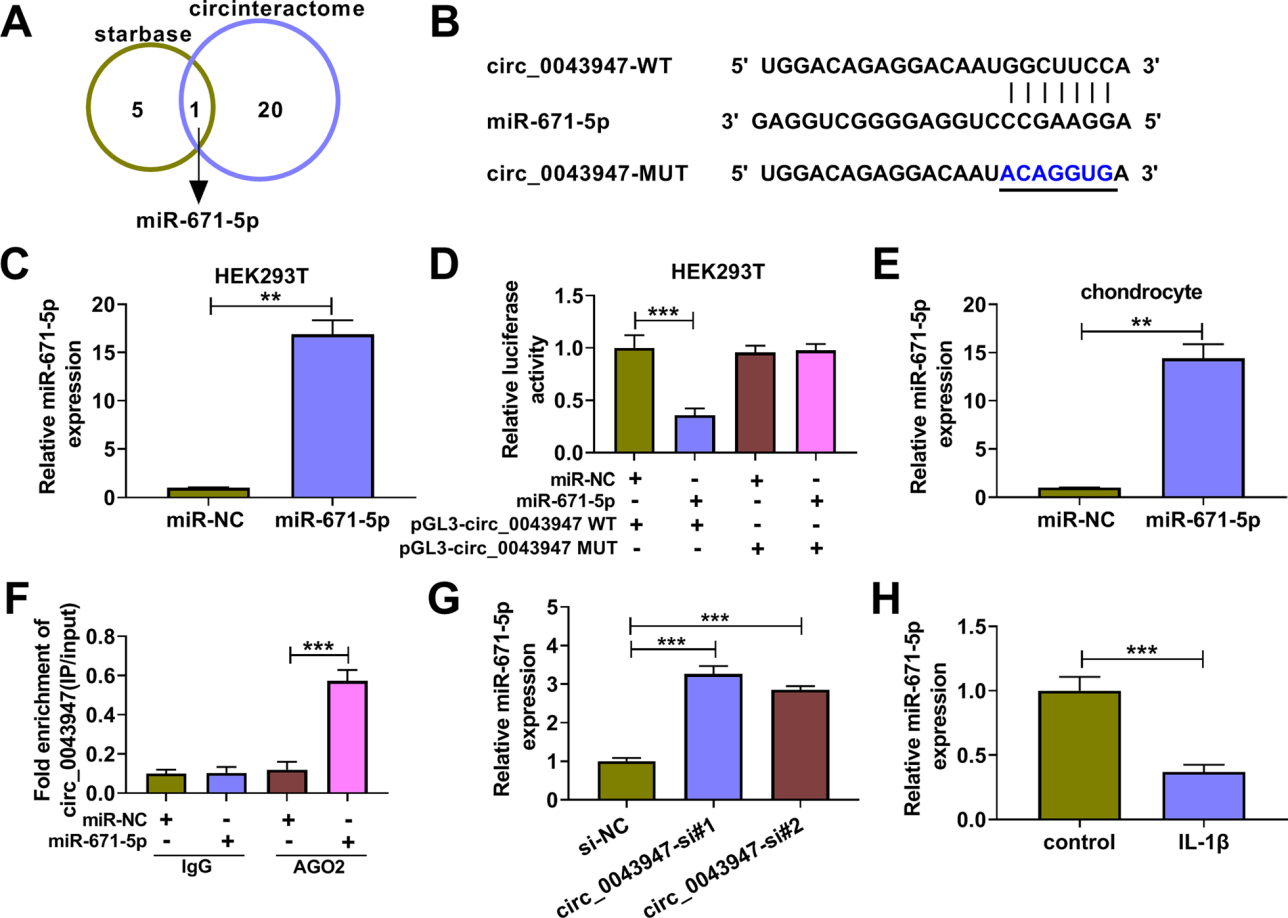
Circ_0043947 acts as a molecular sponge for miR-671-5p. **A** The miRNA targets of circ_0043947 were predicted by bioinformatics algorithms using starbase and circinteractome databases. **B** The putative binding sites with circ_0043947 in miR-671-5p were shown. **C** Overexpression efficiency of miR-671-5p mimics in HEK293T cells was determined by RT-qPCR. **D** Dual-luciferase reporter assay was applied to verify the binding relation between circ_0043947 and miR-671-5p. **E** RT-qPCR assay was conducted to analyze the overexpression efficiency of miR-671-5p in chondrocytes. **F** RIP assay was performed to confirm the target relation between circ_0043947 and miR-671-5p. **G** The effect of circ_0043947 silencing on the expression of miR-671-5p was analyzed by RT-qPCR assay. **H** The expression of miR-671-5p was examined in IL-1β-induced chondrocytes (10 ng/mL, 24 h) by RT-qPCR assay. **P < 0.01, ***P < 0.001

Based on the prediction of two bioinformatics databases, including starbase and circinteractome, miR-671-5p was a candidate target of circ_0043947 (Fig. 3A). The putative binding sites between circ_0043947 and miR-671-5p are shown in Fig. 3B. High overexpression efficiency of miR-671-5p mimics in HEK293T cells was confirmed by RT-qPCR (Fig. 3C). Dual-luciferase reporter assay was conducted to verify the target relationship between miR-671-5p and circ_0043947 in HEK293T cells. Luciferase activity of wild-type plasmid (pGL3-circ_0043947 WT) was markedly reduced by miR-671-5p overexpression, and luciferase activity of mutant plasmid (pGL3-circ_0043947 MUT) was unchanged by the transfection of miR-671-5p or miR-NC (Fig. 3D), suggesting the target relation between circ_0043947 and miR-671-5p in HEK293T cells. Transfection with miR-671-5p mimics notably increased miR-671-5p level in chondrocytes (Fig. 3E). RIP assay was conducted to further validate the interaction between miR-671-5p and circ_0043947 in chondrocytes. Circ_0043947 was abundantly enriched in AGO2 antibody group by miR-671-5p overexpression (Fig. 3F), indicating the interaction between circ_0043947 and miR-671-5p in chondrocytes. Circ_0043947 silencing notably up-regulated the expression of miR-671-5p in chondrocytes (Fig. 3G). After exposing to IL-1β, miR-671-5p expression was markedly reduced in chondrocytes (Fig. 3H). Taken together, circ_0043947 negatively regulated miR-671-5p expression by binding to it in chondrocytes.

### Circ_0043947 silencing-mediated protective effects in IL-1β-induced chondrocytes are largely based on the up-regulation of miR-671-5p

**Fig. 4:**
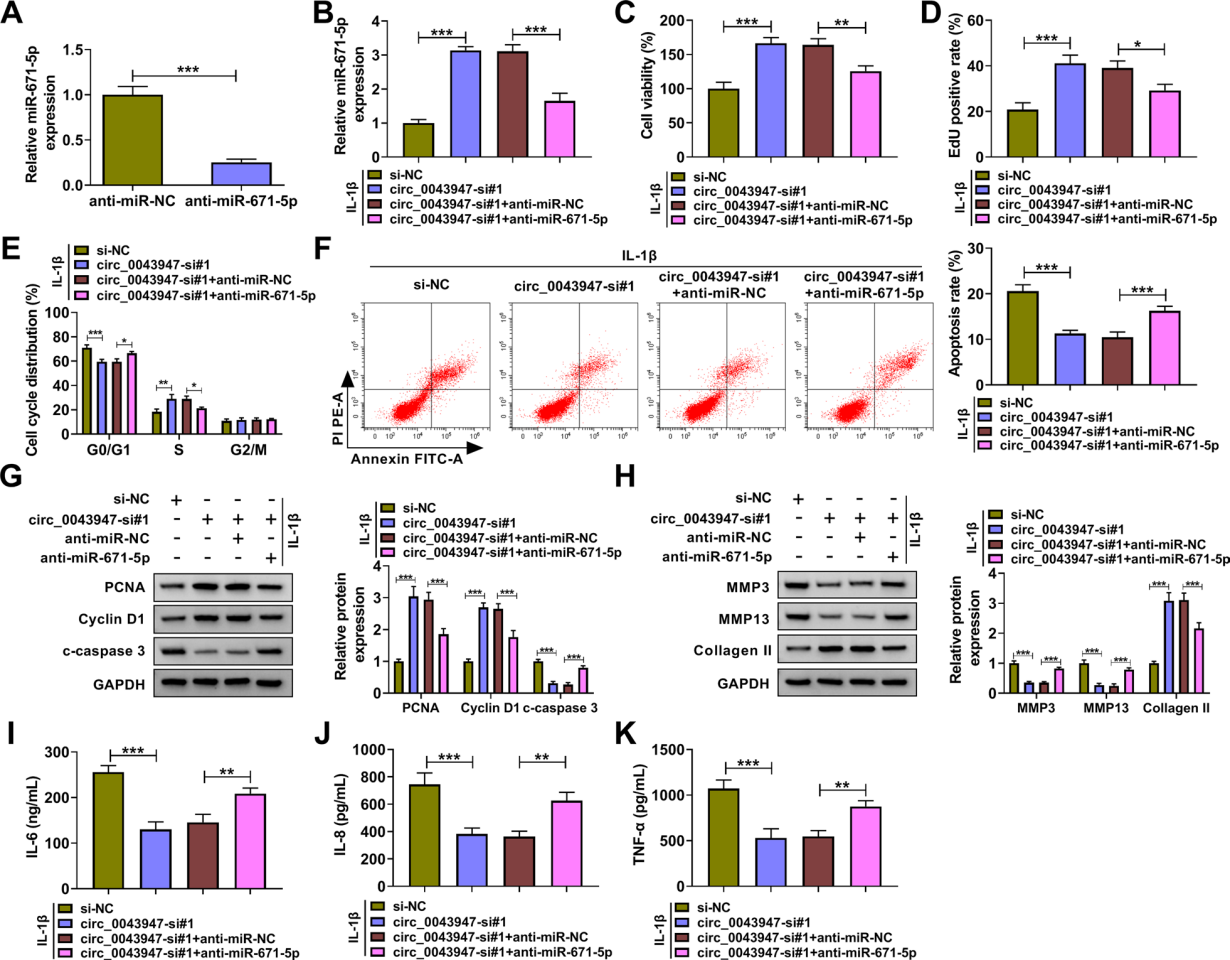
Circ_0043947 silencing-mediated protective effects in IL-1β-induced chondrocytes are largely based on the up-regulation of miR-671-5p. **A** Silencing efficiency of anti-miR-671-5p in chondrocytes was determined by RT-qPCR. **B**-**K** Chondrocytes were transfected with circ_0043947-si#1 alone or together with anti-miR-671-5p prior to IL-1β treatment. **B** The expression of miR-671-5p was examined by RT-qPCR. **C** MTT assay was applied to analyze cell viability of chondrocytes. **D** Cell proliferation was analyzed by EdU assay.**E** and **F** Flow cytometry was applied to analyze cell cycle progression and cell apoptosis. **G** and **H** Western blot assay was implemented to measure the levels of PCNA, Cyclin D1, c-caspase 3, MMP3, MMP13 and Collagen II. **I**-**K** The production of inflammatory cytokines (IL-6, IL-8 and TNF-α) in the culture supernatant was detected via ELISA. *P < 0.05, **P < 0.01, ***P < 0.001

RT-qPCR assay verified the high transfection efficiency of anti-miR-671-5p in chondrocytes (Fig. 4A). To investigate whether the function of circ_0043947 in IL-1β-induced chondrocytes was associated with miR-671-5p, we performed rescue experiments. Circ_0043947 silencing up-regulated miR-671-5p level, and the introduction of anti-miR-671-5p reduced its level again in IL-1β-induced chondrocytes (Fig. 4B). The addition of anti-miR-671-5p decreased cell viability again (Fig. 4C). As verified by EdU assay and flow cytometry, circ_0043947 knockdown-mediated protective effect on the proliferation of IL-1β-induced chondrocytes was largely reversed by miR-671-5p silencing (Fig. 4D, E). miR-671-5p knockdown induced cell apoptosis again in circ_0043947-silenced chondrocytes upon IL-1β exposure (Fig. 4F). Consistent with above results, Western blot assay uncovered that circ_0043947 silencing-mediated effects on the proliferation and apoptosis of IL-1β-induced chondrocytes were largely overturned by the interference of miR-671-5p (Fig. 4G). miR-671-5p knockdown up-regulated the expression of matrix-degrading enzymes (MMP3 and MMP13) and reduced the level of Collagen II (Fig. 4H), suggesting that miR-671-5p silencing induced ECM degradation in circ_0043947-silenced chondrocytes upon IL-1β exposure. Circ_0043947 knockdown suppressed IL-1β-induced inflammation, and cell inflammation was triggered again by miR-671-5p silencing (Fig. 4I–K). These results suggested that circ_0043947 silencing protected chondrocytes against IL-1β-induced effects partly by up-regulating miR-671-5p.

### miR-671-5p interacts with the 3′UTR of RTN3

**Fig. 5:**
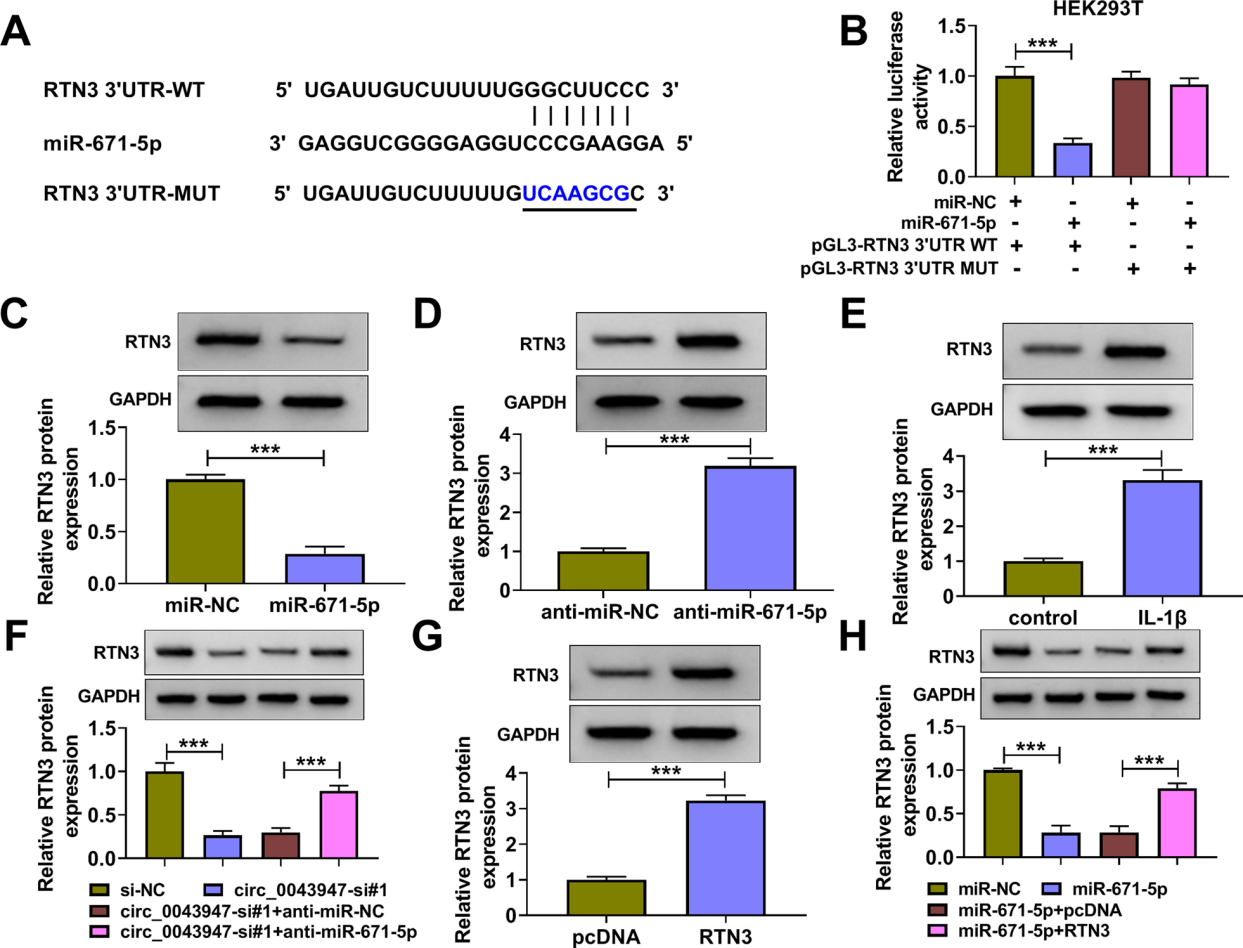
miR-671-5p interacts with the 3′UTR of RTN3. **A** The predicted binding sequence with miR-671-5p in RTN3 was predicted by starbase database. **B** Dual-luciferase reporter assay was conducted to validate the target interaction between miR-671-5p and RTN3. **C** and **D** The effect of miR-671-5p overexpression or knockdown on the expression of RTN3 protein was determined by Western blot assay. **E** The protein level of RTN3 was measured in chondrocytes treated with IL-1β (10 ng/mL, 24 h) by Western blot assay. **F** Chondrocytes were transfected with circ_0043947-si#1 alone or together with anti-miR-671-5p, and the protein level of RTN3 was measured by Western blot assay. **G** The overexpression efficiency of RTN3 plasmid in chondrocytes was determined by Western blot assay. **H** Chondrocytes were transfected with miR-671-5p alone or together with RTN3 plasmid, and the protein expression of RTN3 was measured by Western blot assay. ***P < 0.001

Through bioinformatics starbase database, RTN3 was predicted as a potential target of miR-671-5p (Fig. 5A). Dual-luciferase reporter assay was conducted to confirm the interaction between miR-671-5p and RTN3. Transfection with miR-671-5p markedly reduced luciferase intensity of wild-type luciferase plasmid (pGL3-RTN3 3′UTR WT) rather than mutant type plasmid (pGL3-RTN3 3′UTR MUT) (Fig. 5B), indicating the target interaction between miR-671-5p and RTN3. miR-671-5p overexpression reduced RTN3 protein level, and miR-671-5p silencing up-regulated RTN3 level (Fig. 5C, D), suggesting the negative regulatory relation between miR-671-5p and RTN3 in chondrocytes. After treating with IL-1β, RTN3 expression was markedly elevated in chondrocytes (Fig. 5E). Circ_0043947 knockdown reduced RTN3 expression, and RTN3 protein level was largely recovered by the addition of anti-miR-671-5p in chondrocytes (Fig. 5F), suggesting that circ_0043947 acted as a positive regulator of RTN3 by sponging miR-671-5p in chondrocytes. High overexpression efficiency of RTN3 plasmid was confirmed by Western blot assay (Fig. 5G). miR-671-5p overexpression decreased the protein expression of RTN3, and the addition of RTN3 plasmid largely rescued RTN3 protein level in chondrocytes (Fig. 5H). These findings suggested that RTN3 was a target of miR-671-5p, and it was regulated by circ_0043947/miR-671-5p axis in chondrocytes.

### RTN3 overexpression overturns miR-671-5p overexpression-induced effects in IL-1β-induced chondrocytes

**Fig. 6:**
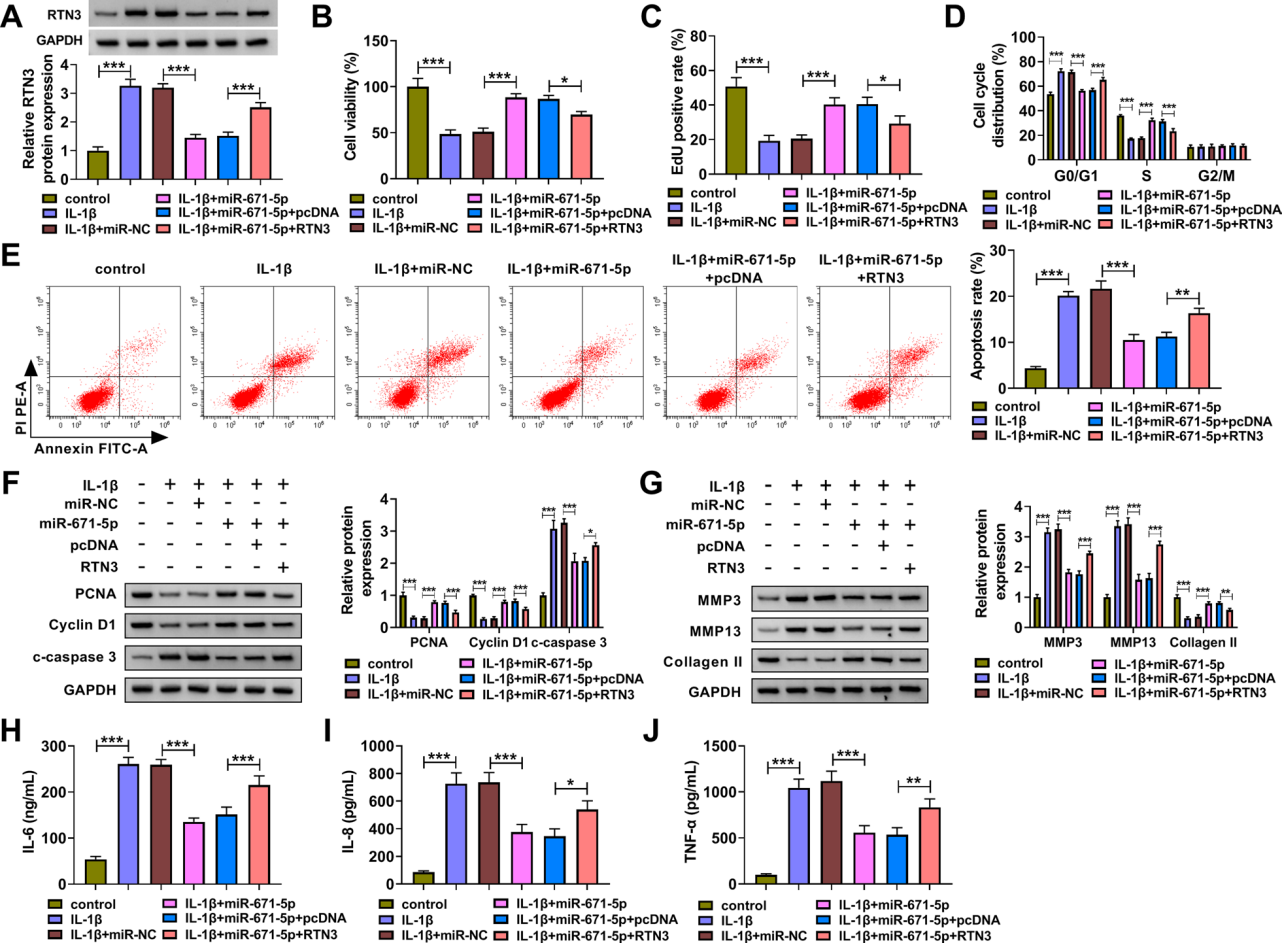
RTN3 overexpression overturns miR-671-5p overexpression-induced effects in IL-1β-induced chondrocytes. **A**-**J** Chondrocytes were treated with IL-1β, IL-1β + miR-671-5p or IL-1β + miR-671-5p + RTN3 and their corresponding controls. **A** Western blot assay was implemented to analyze the protein expression of RTN3 in chondrocytes. **B** and **C** Cell proliferation ability was assessed by MTT assay and EdU assay. **D** and **E** Flow cytometry was utilized to analyze cell cycle progression and cell apoptosis. **F** and **G** The protein levels of PCNA, Cyclin D1, c-caspase 3, MMP3, MMP13 and Collagen II were measured by Western blot assay. **H**-**J** Cell inflammatory response was analyzed by ELISA. *P < 0.05, **P < 0.01, ***P < 0.001

miR-671-5p overexpression reduced RTN3 protein level, and RTN3 protein expression was largely rescued with the addition of RTN3 plasmid in IL-1β-induced chondrocytes (Fig. 6A). miR-671-5p overexpression rescued cell viability and proliferation and suppressed apoptosis, ECM degradation and inflammation in IL-1β-induced chondrocytes (Fig. 6B–J). The addition of RTN3 plasmid restrained the viability and proliferation of miR-671-5p-overexpressed chondrocytes upon IL-1β stimulation (Fig. 6B–D). Cell apoptosis was promoted in RTN3 and miR-671-5p co-transfected group in IL-1β-induced chondrocytes (Fig. 6E). Western blot assay showed that miR-671-5p overexpression protected chondrocytes against IL-1β-induced effects in the proliferation and apoptosis of chondrocytes partly through down-regulating RTN3 (Fig. 6F). RTN3 overexpression increased the levels of MMP3 and MMP13 and reduced the level of Collagen II in miR-671-5p-overexpressed chondrocytes upon IL-1β stimulation (Fig. 6G). Cell inflammatory response was also induced again in RTN3 and miR-671-5p co-transfected group in IL-1β-induced chondrocytes (Fig. 6H–J). These results suggested that miR-671-5p overexpression protected chondrocytes from IL-1β-induced injury partly by down-regulating RTN3.

## Discussion

OA is a chronic degenerative disease induced by pro-inflammatory cytokines such as IL-1β [8]. Many researchers have associated the biological functions of circRNAs with OA pathology [22–24]. For instance, circ-SERPINE2 is reported to restrain OA progression by regulating miR-1271 and ETS-associated molecules [25]. Circ-IQGAP1 silencing is reported to attenuate IL-1β-mediated OA development by targeting miR-671-5p/TCF4 axis [26]. We found that IL-1β stimulation significantly up-regulated circ_0043947 expression in chondrocytes. IL-1β stimulation suppressed the viability and proliferation whereas induced the apoptosis, ECM degradation and inflammation of chondrocytes, and these effects were all largely alleviated by circ_0043947 silencing, indicating that the up-regulation of circ_0043947 was essential for IL-1β exposure-induced injury in chondrocytes.

CircRNAs regulate cellular biological behaviors via their “miRNA sponge” role [27–29]. For instance, circ_33186 is reported to aggravate OA development by serving as miR-127-5p sponge [30]. Circ-CDR1as is reported to contribute to OA pathology by sponging miR-641 [31]. Through starbase and circinteractome databases, we found that miR-671-5p was a possible target of circ_0043947, and their binding relation was then verified by dual-luciferase reporter assay and RIP assay. A negative regulatory relationship between miR-671-5p and circ_0043947 was observed in chondrocytes. Furthermore, we found that IL-1β markedly reduced miR-671-5p expression in chondrocytes. miR-671 is reported to suppress OA progression by previous studies. For example, circ_0114876 is reported to contribute to IL-1β-mediated damage in chondrocytes by up-regulating TRAF2 via acting as a molecular sponge for miR-671 [18], suggesting that miR-671 suppressed OA progression. Zhang et al. found that miR-671 attenuates OA development in vitro and in vivo [17]. Xi et al. found that miR-671-5p is down-regulated in chondrocytes induced by IL-1β, and circ-IQGAP1 contributes to IL-1β-induced OA development by down-regulating miR-671-5p and up-regulating TCF4 [26]. Consistent with these studies, we found that miR-671-5p overexpression protected chondrocytes from IL-1β-induced injury in chondrocytes. In addition, we found that circ_0043947 silencing-mediated effects in IL-1β-induced chondrocytes were largely reversed by the silence of miR-671-5p, demonstrating that circ_0043947 knockdown exerted a protective role in IL-1β-treated chondrocytes partly by up-regulating miR-671-5p.

Reticulons (RTNs) protein family has a typical C-terminal membrane-bound reticulon-homology domain (RHD) [32–34] and is named after its primary subcellular localization in the endoplasmic reticulum [35]. In mouse and human, the RTNs family consists of four members, RTN1 to RTN4. Previous studies have demonstrated that RTNs are implicated in the regulation of neurite outgrowth [36], the activity of Alzheimer’s β-secretase [37, 38], and the pathology of axonopathy in hereditary spastic paraplegias [39]. RTN3 has a role in synaptic plasticity and synapse formation [40, 41], and its role in regulating the neuropathology of Alzheimer’s disease (AD) has been reported [42–44]. However, the biological function of RTN3 in OA pathology is rarely studied. Fu et al. found that LINC02288 facilitates IL-1β-induced apoptosis and inflammatory response in chondrocytes by up-regulating RTN3 via absorbing miR-374a-3p [20], suggesting that RTN3 contributes to OA progression. On the basis of bioinformatics prediction, RTN3 had the potential to bind to miR-671-5p. Then, their interaction was verified by dual-luciferase reporter assay. miR-671-5p negatively regulated RTN3 expression in chondrocytes. After exposing to IL-1β, RTN3 protein level was markedly up-regulated in chondrocytes. To test whether miR-671-5p overexpression suppressed IL-1β-mediated injury in chondrocytes by down-regulating RTN3, we performed rescue experiments. RTN3 overexpression reversed miR-671-5p overexpression-mediated protective role in IL-1β-treated chondrocytes, suggesting that miR-671-5p protected chondrocytes from IL-1β-induced damage partly by down-regulating RTN3. Circ_0043947 acted as a molecular sponge of miR-671-5p to up-regulate RTN3 expression in chondrocytes.

**Fig. 7:**
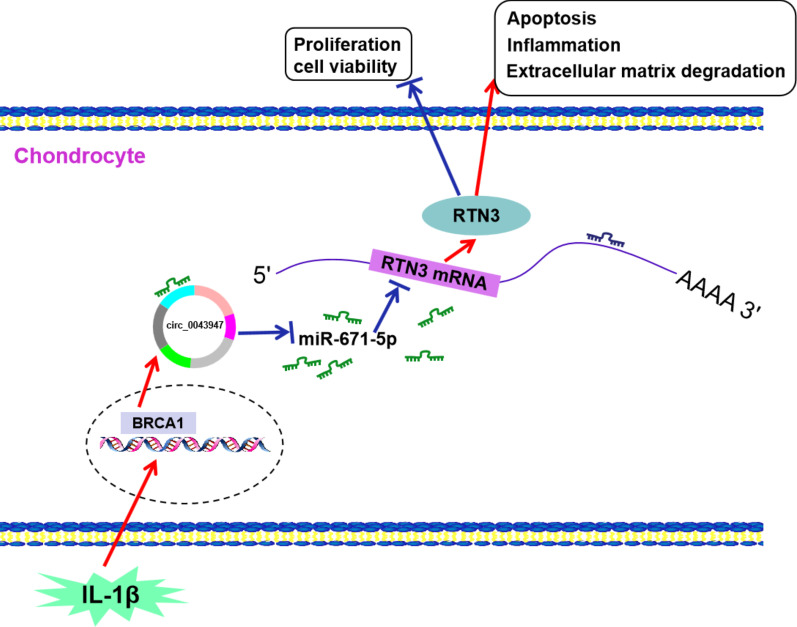
IL-1β suppresses the viability and proliferation whereas induces apoptosis, inflammation and ECM degradation in chondrocytes partly by regulating circ_0043947/miR-671-5p/RTN3 axis

In conclusion, circ_0043947 positively regulated RTN3 expression by sponging miR-671-5p, thereby contributing to IL-1β-mediated effects on the viability, proliferation, apoptosis, ECM degradation and inflammation of chondrocytes (Fig. 7). These results will provide novel potential targets and insight for the intervention and treatment of OA.

## Data Availability

Not applicable.

## Abbreviations

OA: Osteoarthritis
ELISA: Enzyme linked immunosorbent assay
IL-1β: Interleukin 1β
ECM: Extracellular matrix
RTN3: Reticulon 3
MMPs: Matrix metalloproteinases
GAPDH: Glyceraldehyde-phosphate dehydrogenase
